# Extracellular matrix control of dendritic spine and synapse structure and plasticity in adulthood

**DOI:** 10.3389/fnana.2014.00116

**Published:** 2014-10-20

**Authors:** Aaron D. Levy, Mitchell H. Omar, Anthony J. Koleske

**Affiliations:** ^1^Interdepartmental Neuroscience Program, Yale UniversityNew Haven, CT, USA; ^2^Department of Molecular Biophysics and Biochemistry, Yale UniversityNew Haven, CT, USA; ^3^Department of Neurobiology, Yale UniversityNew Haven, CT, USA

**Keywords:** extracellular matrix, dendritic spine, chondroitin sulfate proteoglycans, agrin, reelin, extracellular proteases, RGD peptide, integrins

## Abstract

Dendritic spines are the receptive contacts at most excitatory synapses in the central nervous system. Spines are dynamic in the developing brain, changing shape as they mature as well as appearing and disappearing as they make and break connections. Spines become much more stable in adulthood, and spine structure must be actively maintained to support established circuit function. At the same time, adult spines must retain some plasticity so their structure can be modified by activity and experience. As such, the regulation of spine stability and remodeling in the adult animal is critical for normal function, and disruption of these processes is associated with a variety of late onset diseases including schizophrenia and Alzheimer’s disease. The extracellular matrix (ECM), composed of a meshwork of proteins and proteoglycans, is a critical regulator of spine and synapse stability and plasticity. While the role of ECM receptors in spine regulation has been extensively studied, considerably less research has focused directly on the role of specific ECM ligands. Here, we review the evidence for a role of several brain ECM ligands and remodeling proteases in the regulation of dendritic spine and synapse formation, plasticity, and stability in adults.

## Dendritic spines are highly structured postsynaptic signaling compartments

**Figure 1:**
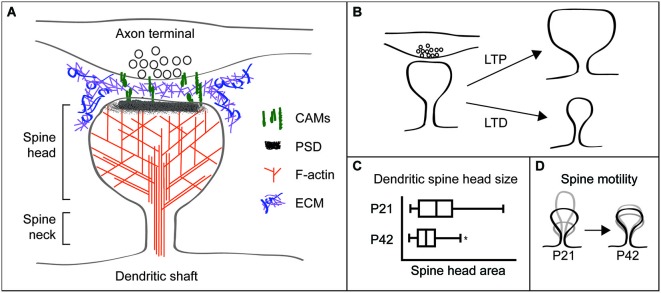
**Dendritic spines are highly structured and plastic synaptic specializations**. **(A)** Schematic of a dendritic spine apposed to a presynaptic terminal. The spine head and neck are supported by a network of filamentous (F)-actin. Postsynaptic cell adhesion molecules (CAMs) connect to the postsynaptic density (PSD) and F-actin in the spine, and extend from the spine to associate with CAMs on the presynaptic terminal. Perisynapic and putative synaptic cleft extracellular matrix (ECM) may interact with multiple CAMs and other cell surface receptors. **(B)** Spine structural changes accompany synaptic plasticity, with long-term potentiation (LTP) increasing spine head size and long-term depression (LTD) decreasing head size. **(C)** Mouse hippocampal CA1 neuron spine head sizes were obtained from electron microscopy of the stratum radiatum. Spine head size and its variance decrease as animals mature from P21 (juvenile) to P42 (adult). Figure modified with permission from Kerrisk et al. (2013). **(D)** Spine motility, defined as changes in spine length over time, is high in juvenile animals. By contrast, spines from adult animals are relatively immotile.

Dendritic spines are protrusions from the dendrite shaft of neurons that comprise the receptive contact at most excitatory synapses in the brain (Gray, 1959a,b; Harris and Kater, 1994; Hering and Sheng, 2001). Ultrastructurally, dendritic spines are composed of a thin neck supported by unbranched filamentous actin (F-actin) and a bulbous head containing a network of branched F-actin (Korobova and Svitkina, 2010; Tønnesen et al., 2014). The spine head also contains the membrane-associated postsynaptic density (PSD), a highly organized network of neurotransmitter receptors, adhesion receptors, scaffolding proteins, and downstream signaling molecules (Harris and Stevens, 1989; Kennedy, 1994, 1997; Hunt et al., 1996; Walikonis et al., 2000; Sheng and Kim, 2011; Harris and Weinberg, 2012). Scaffolding proteins and cell adhesion molecules (CAMs) connect the PSD to the spine actin cytoskeleton. Adhesion molecules also connect to both the presynaptic partner and the extracellular matrix (ECM) in and around the synaptic cleft (Figure 1A). These and other dendritic spine proteins regulate actin filament formation, turnover, and stability, thereby controlling dendritic spine structure.

Spines have a unique structure that is intrinsic to their function. The thin spine neck, ~100–300 nm in diameter (Harris and Stevens, 1989; Tønnesen et al., 2014), restricts diffusion to compartmentalize biochemical and electrical postsynaptic signaling (Majewska et al., 2000; Yuste et al., 2000; Sabatini et al., 2002; Noguchi et al., 2005; Carter and Sabatini, 2008; Harvey et al., 2008; Higley and Sabatini, 2012; Takasaki and Sabatini, 2014; Tønnesen et al., 2014). This compartmentalization enables molecular modifications specific to individual spines and synapses, including changes in synaptic efficacy and spine shape and size. Overall spine head size varies considerably among spines, from ~200–1400 nm in diameter (Harris and Stevens, 1989; Tønnesen et al., 2014). Spine size correlates with synaptic strength and larger spines commonly contain larger PSDs with more AMPA-type glutamate receptors and appose axon terminals with larger readily-releasable pools of neurotransmitter (Harris and Stevens, 1988, 1989; Matsuzaki et al., 2001). Therefore, large spines are more likely to produce strong excitatory postsynaptic currents and have greater influence on neuronal firing and network signaling.

## Dendritic spine structure is dynamic and regulated by activity and development

Recent advances in imaging and single synapse stimulation techniques have revealed that the size and transmission properties of individual dendritic spines can be altered rapidly in response to synaptic activity. Use of glutamate uncaging at individual spines has shown that long-term activity-dependent synaptic strengthening or weakening, also known as long-term potentiation (LTP) and long-term depression (LTD), respectively, occur at discrete synapses and are associated with changes in spine size. High frequency synaptic stimulation that causes LTP promotes spine head enlargement, while low frequency stimulation that causes LTD results in spine head shrinkage (Matsuzaki et al., 2004; Nägerl et al., 2004; Zhou et al., 2004; Oh et al., 2013; Figure 1B). Furthermore, smaller spines are more likely to be lost following LTD-inducing stimulation paradigms (Bastrikova et al., 2008).

Experiments using longitudinal transcranial imaging of individual cortical spines support these *ex vivo* studies. Manipulating sensory input alters the likelihood that dendritic spines will or will not be lost (spine stability) over days, weeks, and months (Oray et al., 2004; Zuo et al., 2005a,b; Lai et al., 2012). Additionally, *in vivo* imaging experiments in mouse models show that stress and genetic abnormalities disrupt normal spine structural dynamics and stability (Pan et al., 2010; Liston et al., 2013). Excitingly, studies using imaging probes that report the activity of specific signaling pathways are beginning to elucidate the signaling events that underlie long-term changes in spine size and signaling properties (Murakoshi et al., 2011; Murakoshi and Yasuda, 2012; Lai and Ip, 2013; Oh et al., 2013; Zhai et al., 2013).

Spine structural plasticity is also heavily influenced by developmental stage. Juvenile animals have increased variance in spine head size (Sfakianos et al., 2007; Kerrisk et al., 2013; Figure 1C) and much more dynamic spine motility relative to spines in adult animals (Dunaevsky et al., 1999; Trachtenberg et al., 2002; Majewska and Sur, 2003; Holtmaat et al., 2005; Figure 1D). Furthermore, higher levels of spine formation and loss occur in adolescent mice vs. adults (Grutzendler et al., 2002).

While the age-dependent loss of spine plasticity has been reproducibly observed, the mechanisms that underlie this phenomenon are not well understood. Multiple synaptic proteins and signaling events differ between juvenile and adult animals as well as between wild type and disease-model animals, which might help to explain differences in spine stability (Scheetz and Constantine-Paton, 1994; Wu et al., 2009; Gundelfinger et al., 2010; Charrier et al., 2012; Akbik et al., 2013; Koleske, 2013). These observations do not, however, directly address whether or how specific pairing of pre- and post-synaptic compartments induces the machinery and mechanisms that confer increased synapse and dendritic spine stability. While it is a difficult experimental question to address, insights into this question are crucial to understanding neurological disorders and how we can gain control of synaptic flexibility.

## Brain disorders involve loss of dendritic spine stability

Loss of dendritic spine stability in adulthood underlies several major neurological and psychiatric disorders, which are accompanied by perceptual, cognitive, memory, and behavioral deficits. For instance, cortical neurons in patients with Alzheimer’s disease, Parkinson’s disease, and other neurodegenerative disorders or dementia have decreased synapse and spine densities (Catalá et al., 1988; Katzman, 1989; Terry et al., 1991; Scheff and Price, 2003). Schizophrenia patients also have reduced cortical spine densities (Garey et al., 1998; Glantz and Lewis, 2000), and medium spiny neurons in Huntington’s disease patients show spine densities that are increased earlier and reduced later in disease progression (Ferrante et al., 1991). Whether spine loss causes disease or results from other problems is unknown, but disrupted network connectivity via spine loss may underlie the cognitive deficits that occur in these patients. These observations demonstrate the importance of dendritic spine stability for normal brain function and suggest that a deeper comprehension of spine stabilization mechanisms could lead to a better understanding of these diseases and possibly new therapeutic approaches.

## Extracellular matrix receptors control dendritic spine stability and remodeling

Several studies demonstrate that specific ECM receptors can regulate dendritic spine stability and remodeling. Brain ECM is composed of secreted proteins and proteoglycans that assemble into cross-linked meshworks to provide structural support to the surrounding cells (Barros et al., 2011; Dansie and Ethell, 2011; Wlodarczyk et al., 2011; Soleman et al., 2013). The brain ECM forms a gel that surrounds neurons and glia, including the space adjacent to and between synapses (Nicholson and Syková, 1998). There, pre- and postsynaptic CAMs associate with one another and with the ECM to initiate and maintain synaptic contact (Bukalo and Dityatev, 2012; Missler et al., 2012). These transmembrane cell adhesion proteins connect to the intracellular dendritic spine actin network and influence the activities of actin regulatory molecules, thereby controlling spine shape (Huntley et al., 2002; Washbourne et al., 2004; Lin and Koleske, 2010; Benson and Huntley, 2012; Cheadle and Biederer, 2012; Sloniowski and Ethell, 2012; Koleske, 2013). Many adhesion molecules also influence synaptic transmission, a key regulator of spine structure (Chan et al., 2006, 2007; Huang et al., 2006; Shi and Ethell, 2006; Bukalo and Dityatev, 2012).

Integrin adhesion receptors are a major family of ECM receptors. Engagement of ECM by integrins triggers changes in cell morphology and motility powered by actin cytoskeletal rearrangements in diverse cell types (Horwitz et al., 1986; Tamkun et al., 1986; Otey and Burridge, 1990; Tawil et al., 1993; Wang et al., 1993; Chong et al., 1994; Gumbiner, 1996; Schwartz and Horwitz, 2006; Schwartz, 2010). Integrins are crucial in the brain as well, where they mediate processes such as migration, axonal outgrowth and pathfinding, and synaptic plasticity (DeFreitas et al., 1995; Chan et al., 2006, 2007; Shi and Ethell, 2006; Belvindrah et al., 2007a,b). Integrin signaling also modulates spine head size and stability during adolescence in mice (Warren et al., 2012; Kerrisk et al., 2013).

Other ECM receptors also function in the brain during adulthood, where they may stabilize spines. For example, dystroglycan, part of the dystrophin glycoprotein complex, plays important roles in axonal pathfinding (Wright et al., 2012) and synapse formation (Sato et al., 2008), but also associates with mature inhibitory synapses and modulates synaptic plasticity (Lévi et al., 2002; Satz et al., 2010; Pribiag et al., 2014). ApoER2, a receptor for the ECM protein reelin, is expressed from late embryonic periods through adulthood, where it is essential for proper migration of cortical neurons in development (Hack et al., 2007) but also plays roles in synapse maintenance and plasticity (Beffert et al., 2005, 2006; Trotter et al., 2011). These and other ECM receptor studies provide strong evidence that ECM regulates dendritic spine stability and remodeling.

## Studying ECM-mediated control of spine structure poses unique difficulties

**Figure 2:**
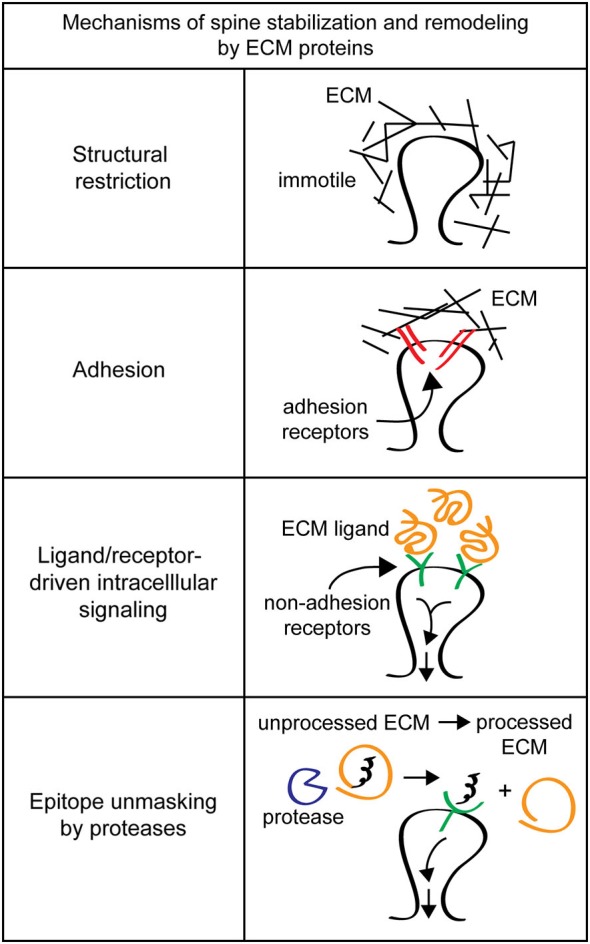
**Mechanisms of spine stabilization and remodeling by extracellular matrix (ECM) proteins**. Extracellular matrix components can stabilize and remodel dendritic spines by a variety of different mechanisms. *Structural restriction*: ECM components such as chondroitin sulfate proteoglycans (CSPGs) can form a matrix around dendritic spines to provide extracellular rigidity and physically restrict spine motion. *Adhesion*: classical ECM proteins such as fibronectin and RGD-containing proteins can act as adhesion substrates and bind to integrin adhesion receptors to remodel spines. *Ligand/receptor-driven intracellular signaling*: ECM proteins like reelin function as ligands for non-adhesion receptors to drive intracellular signaling cascades that regulate spine remodeling and formation. *Epitope unmasking by proteases*: extracellular proteases such as tissue plasminogen activator (tPA) and the matrix metalloproteinase (MMPs) can cleave ECM proteins to reveal cryptic ligands that drive intracellular signaling to change spine morphology.

Extracellular matrix molecules at synapses are likely candidates for regulators of synapse and dendritic spine stability. While studies have identified ECM receptors important for neuronal function and dendritic spine morphology, they often fail to identify the critical ECM ligands that drive these important processes. This failure may be partially due to the inherent difficulty of studying ECM components. Extracellular matrix molecules are secreted, so in the brain where many different cell types are intermingled, cell origin and site of function can be difficult to identify. The heterogenous cell population in the brain also complicates purification of ECM molecules from specific cell types. Additionally, many ECM molecules are large, such as laminins (800 kDa), and can have multiple interaction domains from the same molecule driving distinct pathways (Colognato and Yurchenco, 2000). Extracellular matrix biochemical activities are also altered by covalent modification and/or proteolytic processing, which can be triggered by synaptic activity (Nedivi et al., 1993; Qian et al., 1993; Sung et al., 1993; Szklarczyk et al., 2002; Chen et al., 2008; Horejs et al., 2014). Furthermore, it can be difficult to disentangle possible functions of ECM components in spine stabilization, e.g., providing extracellular rigidity, mediating spine-ECM adhesions, and/or inducing intracellular signaling cascades (Figure 2).

Regardless of the challenges posed by studying the roles of ECM in dendritic spine and synapse stability, emerging evidence indicates that specific ECM components are key regulators of dendritic spine and synapse structure, plasticity, and stability. Here, we review the evidence that specific ECM components and their interaction partners control dendritic spine and synapse structure and how remodeling of the ECM may contribute to dendritic spine plasticity and stability in adults.

## ECM proteins are key regulators of dendritic spine and synapse stability and remodeling

### Chondroitin sulfate proteoglycans restrict functional plasticity and stabilize spines

Chondroitin sulfate proteoglycans (CSPGs), including the lecticans (aggrecan, neurocan, versican and brevican), phosphacan, and leucine-rich CSPGs, are major components of the mature brain ECM. Each CSPG consists of a multi-domain protein core, important for interactions with other ECM molecules, as well as multiple glycosaminoglycan (GAG) side chains that can be degraded by the bacterial enzyme chondroitinaseABC (chABC). The GAG chains are critical for many CSPG functions (Galtrey and Fawcett, 2007), and the pattern of sulfation can define the specific response of the CSPG to signaling partners (Gama et al., 2006). Some CSPGs, notably brevican (Yamada et al., 1994), also exist in non-proteoglycan forms, and loss of CSPG protein core genes is associated with neurological disease (Cichon et al., 2011; Mühleisen et al., 2012). Many CSPGs assemble to form dense peri-neuronal nets (PNNs) around inhibitory neurons (Kwok et al., 2011), which can be identified by staining for GAG epitopes. In addition, a subset of excitatory neurons are also surrounded by more diffuse CSPGs (Wegner et al., 2003).

While the role of specific CSPG core proteins in dendritic spine structure and plasticity has not been extensively studied, a wealth of evidence indicates that the GAG chains of CSPGs restrict circuit plasticity *in vivo*, particularly in the visual system. In the primary visual cortex of rodents and other mammals, cells that receive geniculocortical inputs representing both eyes typically respond more strongly to stimulation of one eye, a phenomenon called ocular dominance (OD). Monocular deprivation (MD) enforced by closing one eye increases the proportion of cells that respond to stimulation of the open eye while reducing the proportion that respond to the closed eye, but only during a critical period for OD plasticity from P19–P35 (Wiesel and Hubel, 1963; Gordon and Stryker, 1996). Chondroitin sulfate proteoglycan expression in primary visual cortex increases through this critical period, and rearing mice in the dark, which delays critical period closure, also delays the developmental increase in CSPGs. This suggests that CSPG expression may be causally linked to the age-dependent loss of plasticity (Pizzorusso et al., 2002). Indeed, while MD normally cannot induce OD plasticity in adult rats after the critical period, MD can shift OD in adult rats that have had chABC injected directly into primary visual cortex (Pizzorusso et al., 2002). In a similar critical period plasticity paradigm, fear memories can be robustly erased by extinction training only during an early critical period, but chABC degradation of PNNs in the amygdala reinstates the ability to erase fear memories in adult rats (Gogolla et al., 2009). In addition, mice lacking the CSPGs neurocan or brevican have deficits in LTP maintenance without other apparent developmental defects, suggesting a role for CSPGs in adults (Zhou et al., 2001; Brakebusch et al., 2002). These results demonstrate that CSPGs are critical for the functional stability of neuronal circuits *in vivo*.

**Figure 3:**
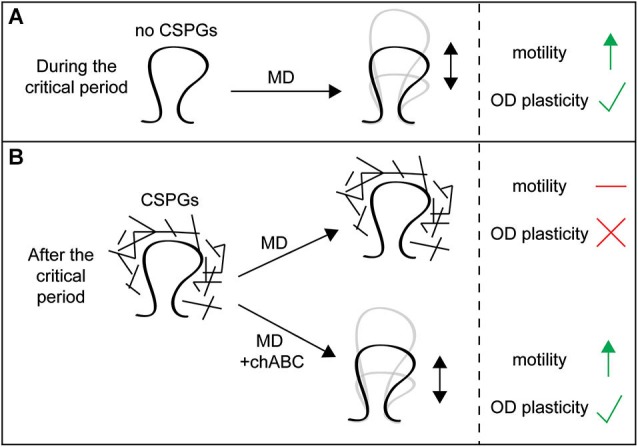
**Chondroitin sulfate proteoglycans around spines restrict spine dynamics and functional plasticity**. **(A)** In juvenile animals during the critical period, CSPG expression is low and visual monocular deprivation (MD) can increase spine motility in primary visual cortex and drive changes in ocular dominance (OD) plasticity. **(B)** In adult animals after the critical period, CSPG expression is high and MD can no longer increase spine motility or drive OD plasticity. However, treatment with chondroitinaseABC (chABC) to degrade CSPG glycosaminoglycan (GAG) chains allows MD to once again increase spine motility and drive OD plasticity in adults, demonstrating that CSPGs restrict spine remodeling and functional plasticity in adult animals.

Chondroitin sulfate proteoglycans normally stabilize dendritic spines. The physiological changes induced by MD are associated with a reduction in spine density of layer II/III visual cortical neurons responsive to the deprived eye (Mataga et al., 2004; Pizzorusso et al., 2006). This loss of spines can be rescued by opening the deprived eye and closing the previously open eye, but only in juvenile animals. However, chABC treatment reinstates this plasticity in adult animals, demonstrating that CSPGs normally stabilize existing spines (Pizzorusso et al., 2006). Loss of CSPGs also enhances spine motility, measured as the magnitude of fluctuations in spine length over time (Figure 1C). Spine motility decreases with age (Majewska and Sur, 2003), but chABC treatment of adult visual cortex *in vivo* and of hippocampal organotypic slices *in vitro* enhances spine motility (Orlando et al., 2012; de Vivo et al., 2013), reverting spines to a more immature phenotype. This is similar to the effect of MD, which also increases spine motility (Oray et al., 2004). These results demonstrate that CSPGs stabilize dendritic spine structure and movement (Figures 3A,B).

Chondroitin sulfate proteoglycans interact with interneurons in PNNs and the development of inhibitory circuits is associated with closure of the critical period, suggesting the effects of chABC treatment on OD plasticity and spine stability may reflect alterations of inhibitory circuits (Pizzorusso et al., 2002). However, emerging evidence indicates that CSPGs can also act directly on spines, independently of PNNs and GABAergic neurons. Orlando et al. (2012) have shown that microinjection of chABC into the stratum radiatum of hippocampal slices, which lacks PNNs but has diffuse CSPG staining, increases CA1 pyramidal neuron spine motility and the number of spines with outgrowths from their heads, mimicking the effects of chABC bath application. This demonstrates that CSPGs normally stabilize spine structure and reduce spine head outgrowths independently of PNNs and inhibitory function (Orlando et al., 2012). These increases in both motility and spine head outgrowth with chABC application require β1 integrin function, which has been shown to be involved in dendrite and spine stability (Warren et al., 2012). While CSPGs interact with and inhibit integrin function (Wu et al., 2002; Tan et al., 2011), whether these specific interactions regulate spine stability is unclear, and should be the target of future studies.

### RGD peptides, and possibly fibronectin, regulate dendritic spine remodeling

In the brain, there is little expression of most of the fibrous ECM proteins such as fibronectin, vitronectin, and the collagens that are major ECM components in other tissues (Ruoslahti, 1996a). Fibronectin mRNA and protein can be detected at low levels in discrete populations of neurons and astroglia, and its expression is increased in the hippocampus of adult animals by kainic acid treatment (Hoffman et al., 1998), but very little is known about the function of fibronectin in the brain. Instead, researchers have more commonly used synthetic peptides common to fibronectin and other matrix proteins that carry an arginine-glycine-aspartate (RGD) motif critical for binding to integrins and for adhesion (Ruoslahti and Pierschbacher, 1987; Ruoslahti, 1996b). RGD peptides have also been shown to evoke changes in synaptic plasticity and structural stability. For example, RGD peptides block the maintenance phase of LTP (Staubli et al., 1990; Bahr et al., 1997; Chun et al., 2001) and potentiate NMDA receptor (NMDAR) currents (Lin et al., 2003; Bernard-Trifilo et al., 2005), demonstrating that RGD-containing proteins may be involved in adult plasticity. RGD peptides also regulate structural stability in mature neurons, as treatment of 14 DIV cultured hippocampal neurons with RGD peptides induces an elongation of existing spines and causes filopodia formation. These changes can be blocked by NMDAR and CaMKII antagonists, suggesting that integrins regulate the stability of dendritic spines via NMDARs and CaMKII *in vitro* (Shi and Ethell, 2006). To be clear, studies using RGD peptides do not demonstrate that any specific RGD-containing ECM protein functions in the brain, as many extracellular proteins have RGD motifs, but they strongly suggest the involvement of integrin receptors in these diverse processes. In addition to traditional “outside-in” integrin activation described above, integrin adhesion can be activated by intracellular signaling partners in an “inside-out” mechanism (Calderwood, 2004; Moser et al., 2009). Inside-out signaling is active but has not been well studied in neurons, and may help explain changes in integrin-mediated ECM contact with changes in neuronal activity. For example, it has recently been shown that reelin signals through its receptors ApoER2 and VLDLR to promote integrin α5β1 adhesion to fibronectin by an inside-out mechanism to control neuronal positioning during cortical development (Sekine et al., 2012). More work needs to be done in the future to establish which RGD-containing brain proteins have effects on synaptic signaling, plasticity and spine structure, and how these signaling mechanisms interact with inside-out signaling pathways.

### Reelin enhances glutamatergic transmission and plasticity and may stabilize spines

Reelin is a 385 kDa secreted ECM protein that is a key regulator of neuronal migration in development (Tissir and Goffinet, 2003; D’Arcangelo, 2014). However, even after neurons have reached their proper destination, reelin continues to modulate synaptic signaling pathways to control dendritic spine structure and synaptic plasticity. Reelin is expressed by layer I and II GABAergic interneurons, primarily Cajal-Retzius cells (Rodriguez et al., 2000), which project to other cortical layers where they secrete reelin into the ECM. Upon release, reelin surrounds and adheres to dendritic shafts and spines of cortical pyramidal cells (Rodriguez et al., 2000; Pappas et al., 2001), suggesting that it might regulate spine structure (Rodriguez et al., 2000). Indeed, younger (P21-P31) heterozygous *reelin*+/− mice, which have grossly normal neuron positioning but only half the level of reelin (Liu et al., 2001; Pappas et al., 2001), have significantly reduced dendritic spine density and altered spine morphology (Liu et al., 2001; Niu et al., 2008; Iafrati et al., 2014). Interestingly, by adulthood *reelin*+/− mice exhibit only minimal spine density loss compared to wild type, suggesting that compensatory mechanisms promote additional spine development when reelin levels are reduced (Ventruti et al., 2011).

In adult animals, reelin continues to promote synaptic function and regulate spine morphology. Adult *reelin*+/− mice have reduced levels of synaptic signaling molecules (Ventruti et al., 2011) as well as deficits in excitatory postsynaptic responses, LTP, and LTD (Qiu et al., 2006a), while addition of recombinant reelin to hippocampal slices or direct injection into the ventricles significantly enhances hippocampal LTP (Beffert et al., 2005; Pujadas et al., 2010; Rogers et al., 2011). Recombinant reelin also increases NMDA and AMPA currents in cultured hippocampal slices and primary hippocampal cultures (Chen et al., 2005; Qiu et al., 2006b; Groc et al., 2007; Qiu and Weeber, 2007). These results together demonstrate that reelin is both necessary and sufficient for adult plasticity and glutamatergic signaling. Reelin is also sufficient to promote spine remodeling, as postnatal overexpression of reelin in the mouse forebrain increases spine head size as well as the number of spines with multiple synaptic contacts, while leaving spine density unchanged. Turning off this reelin overexpression decreases spine size and density, indicating that in some cases reelin may also interact with spine stability mechanisms (Pujadas et al., 2010). In addition, injection of recombinant reelin into the ventricles of adult mice increases hippocampal CA1 spine density (Rogers et al., 2011), suggesting that reelin may also promote spine formation in adults. Interestingly, injection of reelin leads to an hours-long transient increase in reelin levels (Rogers et al., 2011), while genetic overexpression would cause a constant increase, suggesting the timing and duration of reelin expression may be important for its effect on spines. Together, these results indicate that in different contexts, reelin promotes synaptic transmission and plasticity and modulates spine dynamics and stability. Further work on stability would benefit from a conditional reelin knockout mouse that could be used to test the necessity of reelin for spine stability in adult animals.

Reelin levels also appear to affect disease pathology in humans. For example, reelin expression is reduced approximately 50% in patients with schizophrenia (Impagnatiello et al., 1998; Berretta, 2012) and *reelin* haploinsufficiency in mice causes increased neuron packing density, decreased GAD67 levels, reduced pre-pulse inhibition and loss of dendritic spines, all features associated with schizophrenia pathology (Tueting et al., 1999; Liu et al., 2001). Reelin may also be neuroprotective against Alzheimer’s disease, as it has been shown to interact with soluble amyloid-β42, protect against amyloid-β42-induced spine loss and neuron death in cultured neurons, and reduce amyloid plaque development and memory loss in J20 Alzheimer’s model mice (Pujadas et al., 2014). Together, these data show that reelin, which has important roles in neuron development and positioning, also plays critical roles in late onset diseases after development is complete.

### Agrin promotes filopodia and dendritic spine formation

Agrin is best known for its prominent role in development of the vertebrate neuromuscular junction (NMJ) synapse, where it is deposited by motor neurons to induce acetylcholine receptor clustering in the muscle (Gautam et al., 1996; Glass et al., 1996; Sanes and Lichtman, 2001). Agrin is also widely expressed in the brain, with the highest levels of* agrin* expression coinciding with the peak period of synaptic development (O’Connor et al., 1994; Cohen et al., 1997). Indeed, antisense-mediated knockdown of agrin inhibits synapse development in cultured neurons (Ferreira, 1999; Bose et al., 2000). In contrast to knockdown systems, however, cultured *agrin*−/− neurons do not exhibit synaptic deficits (Li et al., 1999; Serpinskaya et al., 1999), suggesting that compensatory mechanisms may arise in the absence of endogenous agrin (Bose et al., 2000). Interestingly, knockdown of agrin in both mature (McCroskery et al., 2009) and immature (McCroskery et al., 2006) neuronal cultures reduces dendritic filopodia number, and agrin overexpression or clustering in immature cultured rat and mouse hippocampal neurons is sufficient to induce filopodia *in vitro* (Annies et al., 2006; McCroskery et al., 2006). As filopodia are precursors for dendritic spines (Ziv and Smith, 1996), these results support a role for agrin in promoting synapse and spine formation even in mature neurons. Indeed, *agrin*−/− mice in which perinatal lethality is rescued by muscle-specific agrin re-expression exhibit decreased cortical dendritic spine density (Ksiazek et al., 2007).

**Figure 4:**
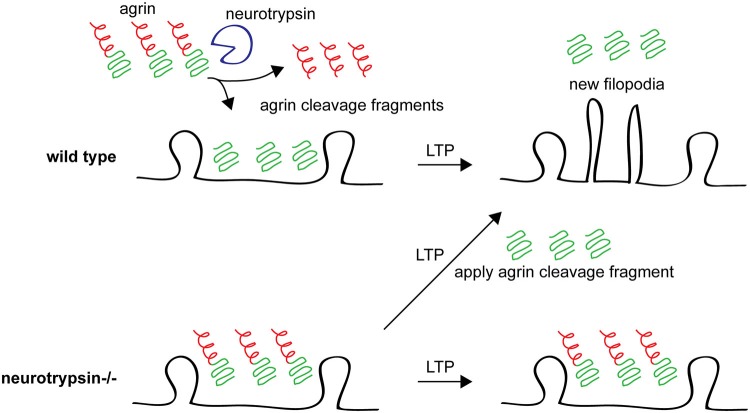
**Agrin cleavage by neurotrypsin plays an important role in filopodia formation following LTP**. In wild type animals after an LTP stimulus, agrin is cleaved by neurotrypsin (top left) and the agrin fragment promotes growth of new dendritic filopodia (top right). In neurotrypsin knockout mice, agrin cannot be cleaved (bottom left) and new filopodia are not formed in response to an LTP-inducing stimulus (bottom right). However, application of a soluble recombinant neurotrypsin-dependent agrin cleavage fragment rescues this phenotype, promoting new filopodia growth after LTP even in neurotrypsin knockout hippocampal slices. See Matsumoto-Miyai et al. (2009).

A role for agrin in spine and synapse stability is also supported by studies of neurotrypsin (also called motopsin or Prss12), an extracellular protease whose only known substrate is agrin (Gschwend et al., 1997; Molinari et al., 2002; Reif et al., 2007; Stephan et al., 2008). *neurotrypsin*−/− mice have reduced CA1 apical dendritic spine density (Mitsui et al., 2009). Neurotrypsin is released from presynaptic neurons in response to NMDAR-mediated activity and cleaves agrin at the synapse, suggesting that neurotrypsin might be involved in activity-dependent plasticity. Indeed, while neurons in hippocampal slices from adult *neurotrypsin*−/− mice have normal electrophysiological LTP, LTP-inducing stimuli fail to induce the formation of new filopodia in the knockouts, suggesting that neurotrypsin is required for some aspects of structural plasticity that accompany LTP in adult animals. Interestingly, a soluble cleavage fragment of agrin produced by neurotrypsin can rescue the loss of LTP-induced filopodia formation in *neurotrypsin*−/− mice (Matsumoto-Miyai et al., 2009; Figure 4). These results suggest a role for neurotrypsin and agrin in supporting new spine formation following LTP induction protocols in mature animals. Further work should address the molecular mechanisms downstream of agrin cleavage that promote filopodia formation and whether and how these new filopodia form functional dendritic spines and synapses.

### Tenascins are required for synaptic plasticity and may interact with spines

The tenascins are a family of ECM proteins that oligomerize through a tenascin association domain and interact with other ECM proteins and receptors through tenascin’s EGF-like and fibronectin type III-repeats (Jones and Jones, 2000). Tenascin R (TNR) and tenascin C (TNC) are both expressed in the brain, TNR exclusively so, where they play roles in synaptic plasticity.

Tenascin R is required for normal plasticity, synaptic transmission, and behavior. Tenascin R knockout mice have impaired hippocampal LTP but normal LTD, increased basal synaptic transmission, and anxiety and motor deficits (Bukalo et al., 2001; Saghatelyan et al., 2001; Freitag et al., 2003; Gurevicius et al., 2004). Tenascin R is mainly associated with CSPGs in PNNs around inhibitory interneurons (Brückner et al., 2000), where it crosslinks some CSPG family members (Aspberg et al., 1997). Tenascin R’s affect on LTP is due to it carrying the human natural killer-1 (HNK1) carbohydrate epitope (Kruse et al., 1985) which normally interferes with *γ*-aminobutyric acid type B GABA_B_ receptor function. GABA_B_ receptors block GABA_A_ receptor-mediated inhibition by reducing presynaptic GABA release through a retrograde mechanism (Saghatelyan et al., 2001, 2003). Therefore the loss of HNK1 with *tenascin-R*−/− disinhibits GABA_B_ receptors, allowing them to block GABA_A_-mediated inhibition and increase excitatory transmission (Saghatelyan et al., 2000, 2001; Nikonenko et al., 2003), raising the threshold for LTP induction (Bukalo et al., 2007).

A key role for TNR in spine stability has been described in the GABAergic granule cells of the olfactory bulb, which have non-conventional dendritic spines that serve both pre- and post-synaptic functions. Tenascin R is expressed and deposited in the olfactory bulb only in adults. Granule cells born in adult *tenascin-R*−/− mice have reduced spine density and reduced migration to the olfactory bulb, while granule cells born in juvenile animals are normal. The reduction in spine density is not secondary to migration defects, as interfering with TNR function after wild type adult-born granule cells have migrated to the olfactory bulb produces a similar reduction in spine density (David et al., 2013). These results demonstrate that TNR regulates the strength of inhibitory contacts onto excitatory neurons to alter adult synaptic plasticity and also regulates spine stability on a subset of inhibitory interneurons.

Tenascin C plays a role in modulating hippocampal plasticity. Tenascin C expression is high early in development but decreases through adolescence and is very low in adults (Ferhat et al., 1996). However, TNC expression can be transiently induced in adult animals by stimuli that cause LTP (Nakic et al., 1998), suggesting a role for TNC in plasticity. Indeed, *tenascin-C*−/− mice have reduced hippocampal CA1 LTP and lack CA1 LTD, though LTP in other regions of the hippocampus is normal (Evers et al., 2002; Strekalova et al., 2002). The specific role for TNC in neuron structure has been understudied, although one study suggests that TNC knockout causes redistribution of stubby dendritic spines in cortex away from primary dendrites and toward higher order dendrites (Irintchev et al., 2005). Further studies will undoubtedly reveal more detailed functions for TNC in spine formation, plasticity, and stability.

### Laminins organize and maintain synapses

Laminins are large, secreted, heterotrimeric glycoproteins made up of alpha (α), beta (β), and gamma (γ) subunits that interact with numerous transmembrane proteins, including integrin receptors, α-dystroglycan, and basal CAM/Lutheran (Horwitz et al., 1985; Buck and Horwitz, 1987; Smalheiser and Schwartz, 1987; Gehlsen et al., 1988; Ignatius and Reichardt, 1988; Gee et al., 1993; Henry and Campbell, 1996; El Nemer et al., 1998; Kikkawa et al., 2007; Aumailley, 2013; Yousif et al., 2013). Multiple α, β, and γ genes have been identified and they can combine to form over a dozen distinct heterotrimers (Aumailley et al., 2005; Aumailley, 2013). Each of the three subunits of laminin have an N-terminal short arm region, which mediates interactions with transmembrane receptors and other ECM molecules, and a coiled-coil domain, which mediates heterotrimerization. The α subunits also have a C-terminal globular domain that engages with cell surface receptors, including several integrins and α-dystroglycan (Colognato and Yurchenco, 2000; Aumailley, 2013).

Early experiments revealed that laminins can promote neurite growth from various cultured neuronal cells (Manthorpe et al., 1983; Liesi et al., 1984; Lander et al., 1985). Subsequently, Sanes and colleagues identified key roles for laminins at the NMJ where specific laminin subunits control development, maturation, and stability of the synapse (Sanes, 1982; Hunter et al., 1989a,b; Martin et al., 1995; Patton et al., 1997, 2001; Nishimune et al., 2008; Samuel et al., 2012). For example, β2 subunit-containing laminins are produced by the muscle and localize to the center of the synapse to direct acetylcholine receptor clustering (Martin et al., 1995). Additionally, an interaction between the β2 subunit and a presynaptic voltage-gated calcium channel maintains active zone organization (Nishimune et al., 2004). The α4 and α5 laminin subunits also play roles at the NMJ where they signal through the ECM receptor dystroglycan to promote postsynaptic maturation. The combined loss of laminins α4 and α5 results in smaller, much less elaborate synapses, and loss of α5 alone from the muscle causes a delay in synapse maturation (Nishimune et al., 2008). Interestingly, loss of laminin α4 also causes premature aging at the NMJ, accelerating age-related phenotypes by several months (Samuel et al., 2012). This work and additional evidence in the peripheral nervous system established laminins as major players in synapse formation and maintenance.

Recent evidence also supports roles for laminins in maintaining synapse structure and stability in the central nervous system. Mice lacking the laminin β2 subunit have disrupted hippocampal synapse structure, including misaligned pre- and postsynaptic partners and increased PSD length (Egles et al., 2007). Co-culturing experiments indicate that β2 laminin is produced by postsynaptic neurons in this system. In the hippocampus, kainic acid injection to induce excitotoxic injury degrades laminin γ1 and causes neuron death. These effects are absent in mice lacking the protease tissue plasminogen activator (tPA) and can be blocked with inhibitors of plasmin, an extracellular protease that is the substrate of tPA and degrades laminins. Importantly, adding a laminin antibody to disrupt laminin-neuron interactions can restore neuronal sensitivity to excitotoxic insult in tPA-deficient mice (Chen and Strickland, 1997). Furthermore, plasmin-mediated laminin degradation is associated with reduced LTP (Nakagami et al., 2000), although specific effects on dendritic spine size or stability have not been investigated.

The roles of laminins in the brain are not as well characterized as their roles in the peripheral nervous system. Nonetheless, these observations suggest that laminins function at synapses to maintain neuronal stability and synapse structure and function. More work is necessary to describe functions of specific laminin subunits in the brain as well as the receptors that mediate CNS laminin:neuronal interactions.

### Netrin:DCC signaling regulates spine morphology and LTP

Netrins are laminin-related proteins that play diverse conserved roles in neuronal morphogenesis and stability (Ishii et al., 1992; Serafini et al., 1994, 1996; Barallobre et al., 2000; Adler et al., 2006; Colón-Ramos et al., 2007; DeNardo et al., 2012; Smith et al., 2012). In mice and humans, the netrin family consists of three secreted molecules, netrins 1, 3, and 4, and two membrane-bound, GPI-anchored proteins, netrin G1 and G2. Receptors for secreted netrins include deleted in colorectal cancer (DCC), the UNC5 family of proteins, and specific integrin receptors (Chan et al., 1996; Keino-Masu et al., 1996; Leonardo et al., 1997; Yebra et al., 2003; Stanco et al., 2009). Interestingly, netrins share homology with the short arm regions of the β or γ subunits of laminin (Lai Wing Sun et al., 2011) and netrin 4 binds the short arm of laminin γ1 and γ3 subunits to form netrin:laminin complexes and disrupt laminin:laminin interactions (Schneiders et al., 2007).

Recent work suggests netrin:DCC interactions might regulate synapse structure and function in the brain. Loss of DCC after initial development causes smaller dendritic spine head size and impairs learning and LTP. Also, Netrin-1 and DCC co-fractionate from synapses of mature rats, and DCC is present at spines of CA1 pyramidal neurons in mature (60 DIV) cultured hippocampal slices (Horn et al., 2013). While this suggests that netrin can regulate both spine morphology and synaptic transmission in adulthood, further study is needed to understand the roles of netrins at synapses in the adult CNS.

## ECM proteases regulate spine and synapse stability and remodeling

### Tissue plasminogen activator creates a permissive environment for spine destabilization

Tissue plasminogen activator (tPA) is a secreted extracellular serine protease best known for its role in cleaving and activating plasminogen into the active protease plasmin to prevent blood clots in the circulatory system (Collen, 1999). In the CNS, tPA is expressed and secreted widely (Sappino et al., 1993; Strickland, 2001), though its activity is located primarily in neurons of the hippocampus, amygdala, cerebellum and hypothalamus (Sappino et al., 1993; Baranes et al., 1998; Lochner et al., 2006). Tissue plasminogen activator was first identified as an activity-dependent immediate early gene strongly induced in rat hippocampus after seizures or LTP (Qian et al., 1993), suggesting a role for the protease in adult plasticity. Indeed, *tPA*−/− mice have deficits specifically in hippocampal LTP maintenance (Frey et al., 1996; Huang et al., 1996) with no problems in short term potentiation paradigms like pre-pulse facilitation or early-phase LTP. Tissue plasminogen activator is also sufficient to support late-phase LTP, as genetic overexpression of tPA enhances LTP proportional to the amount of gene overexpression (Madani et al., 1999). Tissue plasminogen activator can also regulate plasticity in other systems, including cerebellar motor learning (Seeds et al., 2003) and striatal LTD (Calabresi et al., 2000), and *tPA*−/− mice are resistant to chemically-induced synaptic potentiation (Huang et al., 1996; Baranes et al., 1998).

Tissue plasminogen activator is a key regulator of dendritic spine stability during plasticity, both in the visual system and in response to stress. Tissue plasminogen activator becomes activated in binocular primary visual cortex during MD, and tPA knockout blocks MD-induced OD plasticity shifts. Importantly, this loss of plasticity can be rescued by recombinant tPA (Mataga et al., 2002), demonstrating a critical role for tPA in OD plasticity. In addition, MD upregulates spine motility, and this effect can be mimicked by direct application of tPA or plasmin to visual cortex. Importantly, the increased motility induced by MD occludes that caused by plasmin application, suggesting that plasmin and MD function in the same pathway to permit MD-induced structural plasticity (Oray et al., 2004). In addition, while 4 days of MD causes spine pruning in visual cortex during the OD critical period, this spine loss is blocked in *tPA*−/− mice, indicating that tPA is also required for spine pruning in response to MD (Mataga et al., 2004). Chronic stress can also cause spine loss in the hippocampus and amygdala. Plasminogen is activated around dendritic spines by chronic stress, and knockout of tPA or plasminogen blocks stress-induced spine loss (Pawlak et al., 2005; Bennur et al., 2007). These results suggest that tPA negatively regulates spine stability and that its activation creates a permissive environment that destabilizes spines and promotes their loss.

One open question in the field is what substrates of tPA promote plasticity in each of these paradigms. Classically, tPA cleaves plasminogen to make the active protease plasmin, which can then degrade ECM targets. Indeed, plasmin can degrade laminin and the CSPG phosphacan to regulate LTP and hippocampal mossy fiber outgrowth (Nakagami et al., 2000; Wu et al., 2000). Plasmin also likely degrades ECM proteins in the MD paradigm, but its exact targets are unknown (Oray et al., 2004). However, it is important to note that tPA certainly has other non-ECM targets in the brain. For example, the loss of late LTP in tPA and plasminogen knockouts is mostly due to reduced proBDNF cleavage to create the mature form of BDNF (Pang et al., 2004). In addition, tPA can cleave the NR1 subunit of the NMDAR to potentiate NMDAR currents (Nicole et al., 2001). It is clear from these varied results that tPA and plasmin target a variety of ECM and non-ECM proteins to regulate synaptic and structural plasticity. It will be important to clearly identify the critical tPA targets in each experimental paradigm to better understand the role of tPA in dendritic spine regulation.

### Matrix metalloproteinases play diverse roles in dendritic spine remodeling in disease, development, and plasticity

Matrix metalloproteinases (MMPs) are a large class of secreted and transmembrane proteases that can degrade many ECM proteins, transmembrane receptors, and other signaling proteins (Visse and Nagase, 2003). Matrix metalloproteinases were initially identified as critical for brain function because the mRNA for TIMP1, an endogenous inhibitor of MMPs, is upregulated in a kainic acid-induced epilepsy model, suggesting an activity-dependent role for MMPs in epilepsy (Nedivi et al., 1993; Rivera et al., 1997; Jaworski et al., 1999). In the kindling model of epilepsy (Morimoto et al., 2004), MMP9 knockout delays seizure onset while MMP9 overexpression speeds onset (Wilczynski et al., 2008). In addition, MMP2 and MMP9 are expressed in neurons and glia and are upregulated by kainate treatment (Szklarczyk et al., 2002), and other MMPs may also be expressed after injury or certain stimulations (Bilousova et al., 2006; Meighan et al., 2006). Importantly, kainate-induced seizures cause hippocampal spine loss that is blocked in *MMP9*−/− mice (Wilczynski et al., 2008).

**Figure 5:**
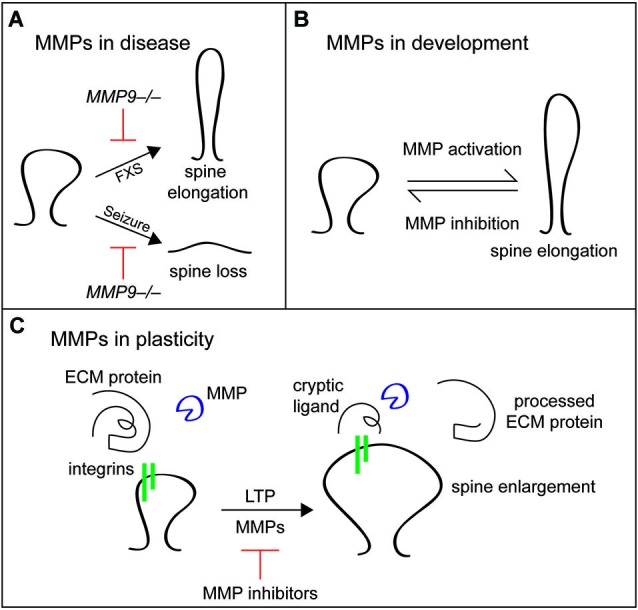
**Matrix metalloproteinases play diverse roles in dendritic spine remodeling in disease, development, and plasticity**. **(A)** In Fragile X syndrome (FXS), MMP9 is hyperactive and dendritic spines are elongated early in development. MMP9 loss of function or inhibition with minocycline normalizes these spine defects. In epilepsy models, seizure-induced spine loss is blocked by inhibition of MMP activity. **(B)** In development, activation of MMPs, particularly MMP9, causes spine thinning and elongation, while MMP inhibition promotes the maturation of filopodia into mature, mushroom shaped spines. **(C)** Matrix metalloproteinases play a different role in adult plasticity, cleaving unknown ECM proteins to reveal cryptic integrin ligands that drive spine enlargement during LTP. In addition to changes in spine size, MMP activity is required for the maintenance phase of LTP.

MMP9 activity is also important in another pathophysiological condition, Fragile X syndrome (FXS). Mice with FXS have more long and thin spines than wild type mice, especially early in development. Treatment of FXS mice or hippocampal cultures derived from these mice with minocycline to inhibit MMP9 can normalize spine morphology (Bilousova et al., 2009), suggesting that hyperactive MMP9 in development prevents spine maturation. Indeed, MMP9 has recently been shown directly to be hyperactive in FXS mice, and disruption of the MMP9 gene in FXS mice normalizes the spine, behavioral, and signal transduction defects associated with FXS (Sidhu et al., 2014). These results demonstrate that pathophysiological activation of MMP9 can promote changes in dendritic spine morphology associated with disease (Figure 5A).

Matrix metalloproteinases also play roles in developmental processes such as spine protrusion and early maturation. For example, bath application of activated MMP9 to young hippocampal cultures or organotypic slices and overexpression of activated MMP9 cause dendritic spines to become longer and thinner (Michaluk et al., 2011). Similarly, treatment of maturing hippocampal cultures with MMP7 causes spine elongation (Bilousova et al., 2006). By contrast, MMP inhibition of cultured neurons promotes maturation of thin filopodial spines into mature mushroom-shaped spines (Tian et al., 2007; Bilousova et al., 2009: Figure 5B). Matrix metalloproteinase activation promotes spine elongation at least in part through cleavage of intercellular cell adhesion molecule 5 (ICAM5). Full length ICAM5 is found in immature neurons and is cleaved by MMPs to release a soluble extracellular domain that promotes filopodial elongation (Tian et al., 2007). Soluble ICAM5 also increases AMPA receptor expression and cofilin phosphorylation, phenotypes that are associated with spine maturation and depend on β1 integrin (Conant et al., 2011; Lonskaya et al., 2013). Interestingly, ICAM5 localization in cortical neurons shifts during synapse development from predominately dendritic filopodia and spines to predominately the dendritic shaft, and this developmental shift is blocked in *MMP9*−/− mice (Kelly et al., 2014). These data indicate that ICAM5 is an important substrate of MMP9 during synaptogenesis. Together, these results show that MMPs have central roles in dendritic spine development and maturation.

It is important to consider that the effects of MMPs on dendritic spines can differ greatly depending on the method of MMP manipulation and the maturity of the system. The MMP-dependent elongation of spines discussed above is dependent on manipulation of MMP activity in young or maturing systems or under pathophysiological conditions and requires general application of MMP-affecting drugs for long periods of time. In more mature systems and with local application of MMPs during plasticity events, MMP activity has the opposite effect. For example, local application of active MMP9 to dendritic spines in acute hippocampal slices is by itself sufficient to potentiate synapses and increase spine volume, the same changes that are caused by theta-burst pairing, which induces LTP. Notably, MMP9-induced potentiation and spine enlargement are occluded by prior theta-burst pairing, suggesting that MMP9 activation and LTP induction function in the same pathway to consolidate spine enlargement and LTP (Wang et al., 2008). Similarly, chemical LTP induction in mature cultured neurons increases spine head size of smaller spines in an MMP-dependent manner (Szepesi et al., 2014). In agreement with these findings, MMP9 is required for maintenance of LTP and LTP-induced spine volume increase in acute hippocampal slices from adult animals (Nagy et al., 2006; Wang et al., 2008), and inhibition of MMPs 3 and 9 blocks acquisition of spatial learning in adult animals (Meighan et al., 2006). Importantly, many of these acute phenotypes in mature systems depend on integrin β1 function (Nagy et al., 2006; Wang et al., 2008), suggesting that MMPs may reveal cryptic integrin ligands in the ECM that maintain spine structural plasticity in mature neurons (Figure 5C). Given the diverse effects of MMP targeting treatments on spine development, plasticity, and maintenance, further studies should address the molecular basis for the differential effects of MMP manipulation in both young and mature systems (Dziembowska and Wlodarczyk, 2012; Stawarski et al., 2014). In addition, the specific ECM molecules that signal through integrins are unknown, and future studies will hopefully link proteolysis of specific proteins by MMPs with specific changes in dendritic spines to understand the signaling mechanisms involved in MMP-mediated dendritic spine remodeling.

## Conclusion

Precise regulation of dendritic spine and synapse formation, plasticity, and stability is essential for proper circuit and brain function. Emerging evidence indicates that ECM proteins, their receptors, and ECM proteases are major physiological regulators of spines and synapses (Table 1). Extracellular matrix molecules are potent regulators of the actin cytoskeleton, which dictates dendritic spine morphology and powers dynamic changes in dendritic spine shape. Moreover, the ECM surrounds neurons and its composition is influenced greatly by synaptic activity, making it an ideal substrate to influence spine and synapse structure and physiology.

**Table 1:** **Role(s) of ECM proteins in synaptic plasticity and/or regulation of spine structure**.

ECM molecule	Role(s) in synaptic plasticity and/or regulation of spine structure	Evidence for role	References
CSPGs	Inhibit adult MD-induced OD plasticity	Degradation of CSPG GAG chains by treatment with chABC permits MD-induced OD plasticity after CP closure in adults.	Pizzorusso et al. (2002)
	Inhibit adult fear memory erasure	Treatment with chABC permits fear memory erasure after CP closure in adults.	Gogolla et al. (2009)
	Required for LTP maintenance	Adult neurocan and brevican knockouts have deficits in LTP maintenance.	Zhou et al. (2001), Brakebusch et al. (2002)
	Inhibit recovery of spine loss due to adult MD	Treatment with chABC allows spine density to recover in adults after MD when the opposite eyelid has been resutured.	Pizzorusso et al. (2006)
	Inhibit spine motility	Treatment with chABC increases spine motility.	Orlando et al. (2012), de Vivo et al. (2013)
RGD peptides	Inhibit LTP maintenance	RGD application to slices or cultured neurons inhibits the late phase of LTP.	Staubli et al. (1990), Bahr et al. (1997), Chun et al. (2001)
	Potentiate NMDA receptors	RGD application increases NMDAR-mediated currents.	Lin et al. (2003), Bernard-Trifilo et al. (2005)
	Increase spine length and promote filopodia formation	RGD application elongates existing spines and induces dendritic filopodia formation.	Shi and Ethell (2006)
Reelin	Enhances LTP	Recombinant reelin enhances LTP magnitude.	Beffert et al. (2005)
	Promotes glutamatergic transmission	Recombinant reelin increases NMDAR and AMPAR currents.	Chen et al. (2005), Qiu et al. (2006b), Groc et al. (2007), Qiu and Weeber (2007)
	Increases spine density	Spine density is reduced in *reelin*+/– mice and enhanced by recombinant reelin.	Liu et al. (2001), Niu et al. (2008), Rogers et al. (2011, 2013), Iafrati et al. (2014)
	Increases spine head size and promotes multi-synapse spines	Recombinant reelin drives these phenotypes.	Pujadas et al. (2010)
Agrin	Promotes filopodia formation	Filopodia formation is promoted by agrin clustering or overexpression and reduced by agrin knockdown.	Annies et al. (2006), McCroskery et al. (2006, 2009)
	Increases spine density	Spine density is reduced in *agrin*−/− and *neurotrypsin*−/− mice.	Ksiazek et al. (2007), Mitsui et al. (2009)
	Required for LTP-induced filopodia formation	LTP-induced filopodia formation is blocked in *neurotrypsin*−/− mice.	Matsumoto-Miyai et al. (2009)
Tenascins	TNR is required for LTP	LTP is impaired in *TNR*−/− mice.	Bukalo et al. (2001), Saghatelyan et al. (2001)
	TNR promotes basal transmission	Basal excitatory transmission is increased in *TNR*−/− mice.	Saghatelyan et al. (2001), Gurevicius et al. (2004)
	TNR required for olfactory bulb granule cell spine density	Spine density of newborn olfactory bulb granule cells is decreased in *TNR*−/− mice.	David et al. (2013)
	TNC is required for LTP and LTD	LTP and LTD are impaired in *TNC*−/− mice.	Evers et al. (2002), Strekalova et al. (2002)
	TNC is required for proper spine distribution along dendrites	Cortical dendritic spines are shifted toward higher order dendrites in *TNC*−/− mice.	Irintchev et al. (2005)
Laminin	Protects against excitotoxicty	Disrupting laminin resensitizes excitotoxic-insensitive neurons.	Chen and Strickland (1997)
	May stabilize LTP	Laminin degradation and loss of LTP are correlated.	Nakagami et al. (2000)
	May be required for synaptic structure	β2 laminin is required for synapse alignment and PSD length.	Egles et al. (2007)
Netrin	May be required for LTP	LTP is impaired in *DCC*−/− mice.	Horn et al. (2013)
	May inhibit spine growth	Spine heads are smaller in *DCC*−/− mice.	Horn et al. (2013)
tPA	Stabilizes LTP late phase	Late LTP is destabilized in *tPA*−/− mice and stabilized by recombinant tPA.	Huang et al. (1996), Frey et al. (1996), Baranes et al. (1998), Madani et al. (1999)
	Required for OD plasticity	OD plasticity is blocked in *tPA*−/− mice.	Mataga et al. (2002)
	Increases spine motility	Spine motility is upregulated by recombinant tPA.	Oray et al. (2004)
	Required for MD-induced spine pruning	Spine pruning caused by MD does not occur in *tPA*−/− mice.	Mataga et al. (2004)
	Require for stress-induced spine loss	Spine loss caused by stress is blocked in *tPA*−/− and *plasminogen*−/− mice.	Pawlak et al. (2005), Bennur et al. (2007)
MMPs	Required for kainate-induced spine loss	Spine loss is blocked in *MMP9*−/− mice.	Wilczynski et al. (2008)
	Required for FXS phenotypes	MMP9 inhibition or deletion rescues spine and behavioral phenotypes in FXS model mice	Bilousova et al. (2009), Sidhu et al. (2014)
	Promote spine elongation	Spine elongation is promoted by MMP activation and blocked by MMP inhibition in young systems.	Bilousova et al. (2006, 2009), Tian et al. (2007), Michaluk et al. (2011)
	Regulate ICAM5 cleavage and function	ICAM5 inhibits spine maturation, and MMPs are required for ICAM5 cleavage to promote spine elongation.	Tian et al. (2007), Conant et al. (2011), Lonskaya et al. (2013)
	Required for LTP late phase	LTP late phase is lost in *MMP9*−/− and *MMP2*−/− mice or when MMPs are inhibited.	Nagy et al. (2006), Wang et al. (2008)
	Required for spatial learning	Morris water maze acquisition is blocked by MMP inhibition.	Meighan et al. (2006)
	Promote LTP-induced spine volume increase	LTP-induced spine volume increase is blocked by MMP inhibition and promoted by local MMP application.	Wang et al. (2008), Szepesi et al. (2014)

*Abbreviations: CSPG-chondroitin sulfate proteoglycans; GAG chains-glycosaminoglycan side chains; MD-monocular deprivation; OD-ocular dominance; LTP-long term potentiation; LTD-long term depression; chABC-chondroitinase ABC; CP-critical period; RGD peptide-Arginine-Glycine-Aspartate peptide; DCC-deleted in colorectal cancer (netrin receptor); tPA-tissue plasminogen activator; TNR/C-tenascin R or C; MMP-matrix metalloproteinase; FXS-Fragile X syndrome*.

Future studies in this field will be critical to identify the molecules that signal through ECM receptors such as integrins to control spine stability and plasticity. Elucidating these molecules and the mechanisms by which they function is essential to understand how differential stability and plasticity are achieved in adulthood vs. development, and in healthy individuals vs. those with neurodegenerative or late-onset psychiatric disease. Only then can we target these mechanisms therapeutically to gain control of synaptic flexibility and stability.

## Conflict of interest statement

The authors declare that the research was conducted in the absence of any commercial or financial relationships that could be construed as a potential conflict of interest.
